# Neuropeptide W

**DOI:** 10.3389/fendo.2012.00171

**Published:** 2012-12-21

**Authors:** Fumiko Takenoya, Haruaki Kageyama, Satoshi Hirako, Eiji Ota, Nobuhiro Wada, Tomoo Ryushi, Seiji Shioda

**Affiliations:** ^1^Department of Anatomy, Showa University School of MedicineTokyo, Japan; ^2^Department of Exercise and Sports Physiology, Hoshi University School of Pharmacy and Pharmaceutical ScienceTokyo, Japan; ^3^Faculty of Health Care, Kiryu UniversityGunma, Japan; ^4^Department of Sports and Health Science, Daito Bunka UniversitySaitama, Japan

**Keywords:** food intake, hypothalamus, brain mapping, peptides, CPCRs

## Abstract

Neuropeptide W (NPW), which was first isolated from the porcine hypothalamus, exists in two forms, consisting of 23 (NPW23) or 30 (NPW30) amino acids. These neuropeptides bind to one of two NPW receptors, either NPBWR1 (otherwise known as GPR7) or NPBWR2 (GPR8), which belong to the G protein-coupled receptor family. GPR7 is expressed in the brain and peripheral organs of both humans and rodents, whereas GPR8 is not found in rodents. GPR7 mRNA in rodents is widely expressed in several hypothalamic regions, including the paraventricular, supraoptic, ventromedial, dorsomedial, suprachiasmatic, and arcuate nuclei. These observations suggest that GPR7 plays a crucial role in the modulation of neuroendocrine function. The intracerebroventricular infusion of NPW has been shown to suppress food intake and body weight and to increase both heat production and body temperature, suggesting that NPW functions as an endogenous catabolic signaling molecule. Here we summarize our current understanding of the distribution and function of NPW in the brain.

## Introduction

**Figure 1:**
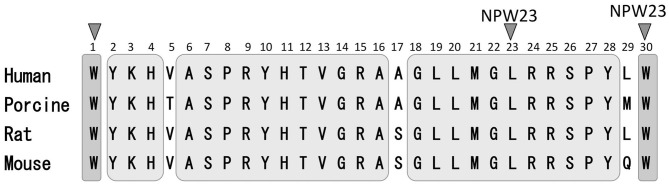
**Sequence comparison of NPW-23 and NPW-30.** Human, porcine rat and mouse NPW-23 and NPW-30 sequences are aligned. A gray area indicates a common amino acid sequence of both NPW-23 and NPW-30.

In 1995, O'Dowd et al. (1995) used oligonucleotides based on the opioid receptor as well as the structurally related somatostatin receptor to identify two genes, GPR7 (NPBWR1) and GPR8 (NPBWR2), which were predicted to encode two G protein-coupled receptors (GPCRs) in the human brain. NPBWR1 mRNA expression is demonstrated in the human and rodent brain, whereas the NPBWR2 gene is detected in the human and rabbit, but not the rodent, brain (Lee et al., 1999). In 2002, Shimomura et al. (2002) identified the endogenous ligand for NPBWR1-2 by exposing Chinese Hamster Ovary (CHO) cells to porcine hypothalamic extracts with monitoring changes in the level of cAMP. Moreover, when cell lines that expressed either NPBWR1 or NPBWR2 were incubated with the hypothalamic extracts, forskolin-induced cAMP production was inhibited. These receptors were coupled to the heterotrimeric Gi protein-mediated receptors (Shimomura et al., 2002). Further structural analysis of the ligands responsible for the inhibition of cAMP production led to the identification of a novel peptide, neuropeptide W (NPW). Shimomura et al. identified mature peptide sequences of both 23 and 30 residues from porcine, rat, and human prepro NPW (Figure 1). NPW is named after the tryptophan residues appearing at both its N- and C-terminals in its two mature forms: NPW30 (the 30 amino acid form) and NPW23 (consisting of 23 amino acids, which are identical to the N-terminal 23 residues of NPW30) (Figure 1). At the same time, the human gene for NPW was identified by Tanaka et al. (2003), who also cloned the mouse gene and reported restricted expression in specific neurons in the mid-brain and brainstem. Similarly, Brezillon et al. (2003) identified the shorter 23 amino acid form of the peptide, which they termed L8 that was derived by proteolytic processing from the longer peptide. The prepropeptide was designated prepro-G protein-coupled receptor 8 ligand.

## Distribution of NPW

Based on RT-PCR analysis, Brezillon et al. (2003) have reported that NPW mRNA is highly expressed in the substantia nigra and spinal cord, and moderately expressed in the hippocampus, amygdala, hypothalamus corpus callosum, cerebellum, and dorsal root ganglia in the human central nervous system. In rodents, *in situ* hybridization histochemistry has revealed that NPW mRNA is expressed in a few restricted brain regions, including the rat periaqueductal gray (PAG), Edinger-Westphal nucleus (EW), and dorsal raphe nucleus (Baker et al., 2003; Tanaka et al., 2003; Kitamura et al., 2006), while Kitamura et al. (2006) have reported that it is confined to specific nuclei in the rat midbrain and brainstem. However, based on RT-PCR analysis, we have reported that NPW mRNA is expressed in the rat hypothalamic paraventricular nucleus (PVN), ventromedial nucleus (VMH), arcuate nucleus (ARC), and lateral hypothalamus (LH) (Takenoya et al., 2010), with another study also reporting the expression of NPW in various areas of the rat brain (Dun et al., 2003). Immunohistochemical studies have shown that NPW-like immunoreactive (NPW-LI) neuronal cell bodies mainly observed in the hypothalamic areas, ARC and posterior pituitary gland, with a lower level in the PVN. Interestingly, NPW-LI cells appear to be more numerous in the male than the female hypothalamus (Dun et al., 2003). In another study, Kitamura et al. (2006) reported a heavy localization of NPW-LI cell bodies in the midbrain, including the PAG and EW. Furthermore, we first identified the presence of NPW-LI cell bodies and their processes in the PVN, VMH, and amygdala at the electron microscopic level (Takenoya et al., 2009). Moreover, NPW-LI nerve fibers were abundantly distributed in the midbrain and limbic system, including the CeA and BST, suggests that NPW may play a role in the regulation of fear and anxiety as well as in feeding behavior (Dun et al., 2003; Hondo et al., 2008; Takenoya et al., 2009, 2010).

In the peripheral tissues, NPW is expressed in the trachea, as well as in lymphoblastic leukemia in the fetal kidney and colorectal adenocarcinoma (Brezillon et al., 2003). Rat adrenocortical cells are also shown to produce NPW (Hondo et al., 2008), as have noradrenalin-containing cells in the rat adrenal medulla (Seki et al., 2008) and gastric antral G cells in rats and mice (Mondal et al., 2006), with expression of NPW in the rat stomach mucosa being regulated by nutritional status, glucocorticoids, and thyroid hormones (Caminos et al., 2008). Hochol et al. (2006) have reported expression of NPW in the thyroid and parathyroid glands, pancreatic islets, adrenal glands, ovary, and testis of the rat, while Rucinski et al. (2007) have demonstrated NPW immunoreactivity in all of the cells of the pancreatic islets, including the A, B, and D cells, and PP cells. In contrast, Dezaki et al. (2008) found NPW immunoreactivity in the B cells, but not in the A or D cells. In addition, NPW mRNA is expressed in the urogenital system, including the kidney, testis, uterus, ovary, and placenta (Fujii et al., 2002). Based on RT-PCR analysis, we have confirmed the presence of NPW mRNA in the pituitary gland, adrenal gland, and stomach (Seki et al., 2008). These observations suggest that NPW may play an important role in the regulation of the endocrine system in response to stress, as well as in the activation of the hypothalamus-pituitary-adrenal (HPA) axis (Niimi and Murao, 2005; Seki et al., 2008).

## Distribution of NPBWR1-2

In humans, RT-PCR analysis has demonstrated that NPBWR1 mRNA is highly expressed in the amygdala, hippocampus, neocortex, and hypothalamus (Lee et al., 1999). *In situ* hybridization histochemical studies have demonstrated that NPBWR1 mRNA is present in the rat hypothalamus, including the ARC, VMH, PVN, and DMH, with Ishii et al. (2003) reported that NPBW1 knockout mice exhibit hyperphagia and develop adult-onset obesity. Singh et al. (2004) used ^[125I]^-NPW receptor autoradiography and demonstrated a significant expression of NPBW1 in the rat amygdala and hypothalamus, as well as in the BST, medial preoptic area (MPA), PAG, superficial gray layer of the superior colliculus, and subfornical organ. In general, NPBWR1 is most commonly expressed at high levels in the amygdala (Singh et al., 2004; Kitamura et al., 2006; Skrzypski et al., 2012). Although the BST shows the highest level of NPBWR1 expression in small mammals, this phenomenon has not been demonstrated in humans. Kitamura et al. (2006) have reported that NPBWR1 is most abundantly expressed in the rat CeA and BST, which may indicate that NPBWR1 is involved in the regulation of stress, emotion, fear, and anxiety. On the other hand, NPBWR1-2 mRNAs are also localized in the pituitary and adrenal glands (in both the adrenal cortex and adrenal medulla) (Mazzocchi et al., 2005). These observations suggest that NPBWR1-2 may be involved in responding to stress via the HPA axis (Mazzocchi et al., 2005; Niimi and Murao, 2005). Ziolkowska et al. (2009) recently examined the expression and function of the NPW, NPB, and NPBWR1 system in cultured rat calvaria osteoblast-like cells, with their results suggesting a direct effect on this cell proliferation. NPB has been identified in larger mammals, as well as in rabbits, but has not been described in either rats or mice. RT-PCR analysis has shown that NPBWR2 mRNA is highly expressed in the human amygdala, hippocampus, pituitary gland, adrenal gland, and testis, as well as in the cortical cells in the adrenal gland (Mazzocchi et al., 2005).

## Regulation of feeding and energy metabolism by NPW

NPBWR1 knockout mice are hyperphagic and show decreased energy expenditure, suggesting that NPW may act as a modulator of feeding. icv infusion of NPW in male rats has been shown to increase food intake during the first 2 h in the light phase (Shimomura et al., 2002). Similarly, Levine et al. (2005) have reported that injection of NPW into the PVN increases food intake. These results suggest that NPW acts as an acute orexigenic peptide. However, Mondal et al. (2003) have reported that both forms of NPW suppress dark-phase and fasting-induced food intake, indicating that the effect of NPW on feeding differs depending on whether animals are maintained in a light or dark phase.

**Figure 2:**
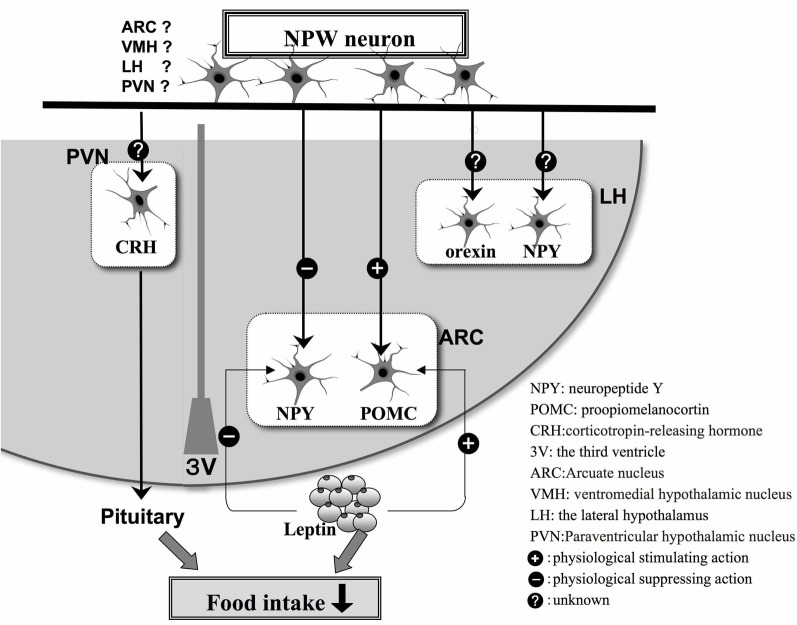
**Schematic illustration based on the findings of morphological and physiological studies of appetite regulation in the hypothalamus by NPW neurons and feeding-related peptides in hypothalamus.** The plus or minus indicates stimulatory (+) or inhibitory (−) effects, respectively.

We have carried out a series of neuroanatomical studies to examine the neural relationship between NPW and other neuropeptides involved in the regulation of feeding. Very close neuronal interactions were observed between NPW-containing nerve fibers and orexin- or melanin-concentrating hormone-containing neuronal cell bodies and nerve fibers in the rat brain (Takenoya et al., 2005), while Levine et al. (2005) demonstrated that c-Fos expression was induced in orexin-containing neurons in the perifornical region of the LH after intracerebroventricular (icv) infusion of NPW. Interestingly, we also identified NPW-LI cell bodies in the VMH, which is known as a center for satiety (Takenoya et al., 2010). Leptin acts on neurons in the VMH, thereby reducing food intake, and Date et al. (2010) have recently reported that NPW-LI neurons and leptin receptors are colocalized in this region of the brain. NPW expression is also significantly up-regulated in *ob/ob* and *db/db* mice. Therefore, NPW may play important roles in feeding and energy metabolism, functioning as a substitute for leptin (Date et al., 2010) (Figure 2). Furthermore, NPW reduces food intake via the melanocortin-4-receptor signaling pathway, suggesting that it may activate POMC-containing neurons and inhibit NPY-containing neurons to control feeding regulation in the ARC (Date et al., 2010) (Figure 2).

Very recently, Skrzypski et al. (2012) have demonstrated that NPB and NPW regulate the expression and secretion of leptin and resistin, and increase lipolysis in isolated rat adipocytes. When NPW was administered to rats, the authors could not detect enhanced locomotor activity, but did observe increased O_2_ consumption and increased CO_2_ production, as well as an increase in body temperature (Mondal et al., 2003). Interestingly, Mondal et al. (2006) have reported that the levels of NPW isolated from rat stomach antral cells are lower in fasted animals, increasing once the animals have been re-fed. In contrast, female NPBWR1 −/− mice do not display hyperphagic activity compared with wild-type mice (Ishii et al., 2003). In addition, Dun et al. (2003) reported the existence of differences between male and female rats with respect to the distribution of NPW.

## Neuroendocrine function of NPW

Immunohistochemical studies have reported that NPBWR1 is expressed in the PVN, pituitary gland, and adrenal medulla in the human, mouse and rat (O'Dowd et al., 1995; Lee et al., 1999; Brezillon et al., 2003; Tanaka et al., 2003), particularly in the parvocellular subdivisions of the PVN and the posterior pituitary. However, NPW has not been reported to influence the release of other anterior pituitary hormones. These neuroendocrine effects of NPW are not directly mediated through NPWBR1 on the pituitary gland cells, but may occur indirectly through controlling the release of the hypothalamic hormone, corticotrophin-releasing factor (CRF) (Brezillon et al., 2003; Samson et al., 2004). Colocalization of NPW with urocortin, a stress-related neuropeptide of the CRF family, has also been demonstrated in the EW.

On the other hand, icv infusion of NPW23 stimulates prolactin release in the rat (Shimomura et al., 2002), and an *in vitro* study has reported that log molar NPW23 concentrations, significantly alter prolactin, growth hormone and ACTH release from dispersed rat anterior pituitary cells (Baker et al., 2003). In addition, *in vivo* studies have revealed that icv infusion of NPW23 stimulates an increase in plasma prolactin levels (Baker et al., 2003). icv infusion of NPW23 also results in a significant, elevation in plasma corticosterone levels, suggesting that NPW plays a role in the hypothalamic response to stress. However, growth hormone levels in plasma are inhibited by icv infusion of this peptide. These findings suggest that NPW is the endogenous ligand for GPR7 and/or GPR8 and acts as a mediator of neuroendocrine function (Shimomura et al., 2002; Baker et al., 2003). Moreover, Taylor et al. (2005) have reported that the infusion of NPW activates the HPA axis, as demonstrated by changes in plasma corticosterone levels in conscious rats; NPW increases plasma corticosterone levels but does not stimulate the release of oxytocin or vasopressin in the peripheral circulation, or alter blood pressure or heart rate. Furthermore, icv infusion of a CRF antagonist does not significantly reduce corticosterone levels, although CRF antagonist pretreatment significantly reduces the capacity of centrally administered NPW to increase corticosterone levels (Taylor et al., 2005). Using electrophysiological studies with whole-cell patch recording of hypothalamic slice preparations, Taylor et al. (2005) have also shown that NPW depolarizes and increases the spike frequency of the majority of putative neuroendocrine PVN neurons. Furthermore, the response of these cells in the presence of tetrodotoxin confirmed that NPW was acting post-synaptically (Taylor et al., 2005).

Niimi and Murao (2005) have reported that double immunostaining for both NPW and c-Fos is significantly increased in response to stress. Immobilization stress increases parasympathetic outflow, whereas cold stress is primarily considered to increase sympathetic outflow, suggesting that NPW may participate in the regulation of both the sympathetic and parasympathetic branches of the autonomic nervous system. In the PVN, vasopressin- expressing neurons are known to be involved in the production of hormonal outputs in response to endocrine and autonomic stress (Ulrich-Lai and Herman, 2009). Kawasaki et al. (2006) have reported that centrally administered NPW activates magnocellular neurosecretory neurons in the SO and PVN, and significantly increases plasma arginine-vasopressin and plasma oxytocin levels. Price et al. (2008) have reported that the inhibition of growth hormone release due to the central effects of NPW results from activation of arcuate somatostatin neurons, which could produce inhibition of neurons expressing growth hormone releasing hormone. These findings suggest that endogenous NPW may play a physiologically relevant role in the neuroendocrine response to stress in the brain (Table 1).

**Table 1:** **Physiological effect of NPW in Rat**.

**Effect**		**References**
Food intake	↑	Shimomura et al. (2002), Levine et al. (2005)
	↓	Mondal et al. (2003)
Body weight	↑	Tanaka et al. (2003)
Heart rate	↑	Yu et al. (2007a)
Arterial blood pressure (ABP)	↑	Yu et al. (2007b)
ACTH	↑	Hochol et al. (2007), Yogo et al. (2012)
Prolactin	↑	Baker et al. (2003), Samson et al. (2004)
Growth hormone	↓	Baker et al. (2003), Samson et al. (2004)
Parathyroid hormone	↑	Hochol et al. (2006)
Corticosterome	↑	Baker et al. (2003), Taylor et al. (2005)
Testosterone	↑	Hochol et al. (2006)
Estradiol	↑	Hochol et al. (2006)

The plus or minus indicates stimulatory (↑) or inhibitory (↓) effects, respectively.

### Conflict of interest statement

The authors declare that the research was conducted in the absence of any commercial or financial relationships that could be construed as a potential conflict of interest.
